# Is Teachers' Depression Contagious to Students? A Study Based on Classes' Hierarchical Models

**DOI:** 10.3389/fpubh.2022.804546

**Published:** 2022-03-21

**Authors:** Wenfeng Wu, Yongbiao Lu

**Affiliations:** The School of Psychology, Guizhou Normal University, Guiyang, China

**Keywords:** depression, emotional contagion, emotional labor, teachers, students

## Abstract

**Background:**

According to the theory of emotional contagion, emotions in one person can trigger similar emotions in groups within social networks. In schools, the class just like a small social network, that teachers' emotion, such as depression, might be contagious to their students. However, until now there is few studies reporting this issue. This study aims to explore whether teachers' depression be contagious to students and what mechanics behind the phenomenon.

**Methods:**

Using Children's depression and cognitive scales to assess 2,579 students, meanwhile using teachers' depression and emotional labor scales assess 529 teachers. The nested data from 112 classes were analyzed.

**Results:**

Teachers' depression was positively correlated with emotional labor surface and deep acting, and teachers' depression cross-level predicted students' depression inversely. For teachers with higher levels of depression, the teacher's deep acting affected their students' depression significantly, the more effortful the teachers' deep acting, the lower the degree of the students' depression, however, for teachers with lower levels of depression, the deep acting was not significant.

**Conclusion:**

The results maybe state that depression in teachers is not readily transmitted to students, one of reasons is that teachers' emotional labor may alleviate the influence of their depression on students. However, considered that teachers' emotional labor was positively correlated with their depression, the teachers' emotional labor may be like a double-edged sword, while alleviating the influence of teachers' depression on students, it also deteriorated their own depression, making it impossible sustainable. For students' depression interventions based in school, including teachers maybe a better selection.

## Introduction

Approximately one half of mental disorders begin in childhood and adolescence (1–3) and the global prevalence of mental health disorders in children and adolescents is almost 15% (4, 5). Epidemiological studies show that depression and anxiety are among the most prevalent and recurrent mental disorders in children and adolescents (6–8). With an estimated 1-year prevalence of 4 ~ 5% in mid to late adolescence, depressive disorder has become one of the common mental health problems in adolescents (9).

In China, we have not found reports concerning nationwide investigation of depression in children and adolescents. However, some regional studies have shown that the prevalence of depressive symptoms is similar to those stated in worldwide reports. For example, studies on the prevalence of depressive symptoms in children yielded figures of 6.4% in the Guangdong province and 13.2% in Beijing city (10, 11). Further studies conducted on the prevalence of depressive symptoms in adolescents found incidences of 15.7% in Nanjing city and 21.9% in the Hunan province (12, 13). A meta-analysis study examined 1,460 Chinese articles concerning the prevalence of depression in Chinese children and adolescents published from 2000 ~ 2014 and selected 14 which included a total of 72,402 participants from 16 provinces (districts or cities). The meta-analysis found that the pooled prevalence of depression was 15.4% (14).

Reports suggest high incidences of depression symptoms in children and adolescents, and that youth depression is associated with numerous adverse personal, social, and academic outcomes (15, 16). Moreover, if left untreated, depressive symptoms tend to persist and increase in severity during adolescence and adulthood (17). Substantial and sustained efforts have been made over many decades to develop and evaluate evidence-based treatment for depression in children and adolescence (18). Besides treatment, researchers have also investigated programs and initiatives designed to prevent depression onset in children and adolescents (19–22). When implementing a prevention program, it is critical to identify the sources of heterogeneity in intervention effects and to attempt to bolster the impact of interventions. One means of achieving this goal is the implementation of prevention programs in schools, for that community surveys of mental disorders in children and adolescents have commonly used schools as a sampling frame (4). Schools have unparalleled contact with children and adolescents and represent a location in which the majority of children and adolescents can be reached. Therefore, schools are considered to be an ideal environment in which to implement prevention programs (21, 23). For this reason, prevention programs based in schools have been increasingly implemented (24–26). Werner-Seidler et al. (26) summarized studies on school-based prevention programs for depression, concluding that: (1) school-based prevention programs have small effects on depression, but do have the potential to reduce mental health burdens; (2) prevention program types and the personnel implementing the prevention program influence the outcomes; and (3) there is some evidence that interventions for depression delivered by external personnel are superior to those delivered by school staff. However, Jennings and Greenberg (27) posited that to ensure children's wellbeing, mental health interventions in schools must begin with mentally healthy teachers. In this regard, Granger's study (28) reported that teachers' wellbeing, specifically teacher depression, limits the extent to which teachers engage in limits the extent to which teachers engage in high-quality interactions with their students. Therefore, it can be hypothesized that teachers' depression is one important variable which affects the outcomes of interventions for students' depression in schools.

Depression is a type of emotional disorder (29) and, according to the theory of emotional contagion, emotions, like infectious diseases, can spread through groups of people in social networks (30). Studies have found the effects of emotional contagion could be long term and profound. Obesity, smoking, alcohol use, depression, loneliness, and happiness can spread through the web of relationships within broader social networks (31). A meta-analysis of 40 results from 36 studies also provided substantial overall support for the proposition that depressive symptoms and mood are contagious (32). In the field of organizational behavior study, after reviewing the related literatures, Tee suggested that leaders' management and regulation of emotional contagion processes underlies the shape, form, and outcome of organization-wide culture, climate and change outcomes (33). Based on the group of students, a teacher's job is similar to that of a leader in an organization. Teachers' mannerisms and emotional states may contribute to the class's emotional atmosphere, as well as the school's atmosphere. Therefore, according to the theory of emotional contagion, we surmise that teachers' depression may be transmitted to their students.

In the standard medical susceptible-infected susceptible (SIS) model, individuals are classified as occupying one of two states: “susceptible”, meaning they do not have the disease, and “infected”, meaning they do have the disease. The disease can be transmitted to a susceptible person when they come into contact with an infected person. That means infection can only be contagious by having a contact of an infected and a susceptible individual (30). Extrapolating from the SIS model, we believe that teachers' depression may more easily transmit to students who are vulnerable to depression. In this regard, an extensive body of research has demonstrated that vulnerable cognitions related to depression contribute to the onset and development in children and adolescents (34–36). We hypothesize that vulnerable cognitions related to depression could have a role in the process of teachers' depression transmitting to students.

In addition, another study found that teachers' emotional labor moderated the influence of stressful life events on students' depressive symptoms (37). Emotional labor is a term first coined by Hochschild (38) which refers to the act of regulating one's emotions to satisfy organizational requirements. Emotional labor has two primary regulation strategies: surface acting and deep acting. Surface acting refers to people presenting the emotional state required by organization, but not experiencing the emotion internally; by contrast, deep acting involves not only displaying an emotional state, but also feeling and experiencing the emotion on a personal level (38, 39). These two emotional strategies would generate different consequences (40). Since a teacher's job requires effort to ensure appropriate emotional expressions during the course of their work, the act of teaching contains typical characteristics of emotional labor (41). We believe that teachers may regulate depressive emotions through emotional labor and that this may affect the transmission or conveyance of teachers' depression to students.

The purpose of this study is to explore whether teachers' depression can be transmitted to their students, and, if so, how is it transmitted and what are its mechanisms? We hypothesize the following: (1) depression in teachers can undergo cross-level transmission to their students in the class group; (2) students' vulnerable cognitions to depression moderate or mediate the relationship between teachers' depression and their students' depression; (3) teachers' emotional labor cross-level moderates the relationship between teachers' depression and their students' depression.

## Methods

### Participants

#### Teacher Samples

We investigated 529 teachers from 112 classes in 11 schools in the Hunan province, China. Among them, 166 were males, 290 were females, and 73 did not indicate their gender. Of the sample, 435 were married, 61 were unmarried, 1 was divorced, and 32 did not indicate their marital status. The average amount of teaching experience was 16.59 ± 8.75 years, the shortest being 1 year and the longest being 49 years. Of the sample, 97 were class head teachers and 15 classes had no information concerning the class head teachers.

#### Student Samples

The total student sample from the 112 classes was 2,579, including 1,212 males (male coded “1”) and 1,256 females (female coded “2”), while 111 did not indicate their gender. Only-children (coded “1”) comprised 1,302 members of the sample while 1,120 had siblings (coded “2”) and 157 did not fill in the relevant information. The average age was 13.35 ± 1.01 years and the study sample included 1,232 students in the 7th grade, 842 in the 8th grade, and the 505 in 9th grade. The average number of students in every class investigated was 23.03 ± 5.47, the minimum number was 9 and the maximum was 36.

All the teachers who participated in the study filled out the informed consent, and all the students who participated in the study filled out the informed consent by their parents.

### Measures

#### Students' Depressive Symptoms

Students completed the Children's Depression Inventory (CDI), which was developed by Kovacs (42) and translated into Chinese (43). The scale consists of 5 dimensions: anhedonia, negative self-esteem, negative mood, ineffectiveness, and interpersonal problems, measured by 27 items. The total score range is 0 54, and higher scores represent a greater severity of depressive symptoms. In this research, the total scale Cronbach'α coefficient is 0.861.

#### Students' Cognitive Vulnerability to Depression

Students completed the Children's Cognitive Style Questionnaire (CCSQ) (44) and Children's Dysfunctional Attitudes Scale (CDAS) (45). The CCSQ contains two parts. In the first portion, participants are given twelve hypothetical negative events and asked to circle which statement best describes their thought process following the event: *(a) This won't cause other bad things to happen to me; (b) This might cause other bad things to happen to me; (c) This will cause other bad things to happen to me; or (d) This will cause many terrible things to happen to me*. Each response is assigned a value from 0 to 3 with higher scores indicating a greater tendency to catastrophize the consequences of a negative event. For the second part of the CCSQ, participants are given a hypothetical, negative event and asked to circle which statement best describes their thoughts following the event: *(a) This does not make me feel bad about myself; (b) This makes me feel a little bad about myself; or (c) This makes me feel very bad about myself*. Each response is assigned a value of 0 to 2, with higher scores representing a tendency to view oneself more negatively following an event. In this study, Cronbach'α coefficient of the first part of CCSQ Questionnaire is 0.785; and the second part is 0.758.

The CDAS is a self-report questionnaire designed to assess dysfunctional attitudes in children and adolescents. For each item, participants are asked to rate how strongly each statement applies to them (i.e., “*never true*,” “*sometimes true*,” “*mostly true*,” and “*always true*”). In this research, the total questionnaire Cronbach'α coefficient is 0.810.

#### Students' Life Events

Students completed the Adolescent Self-Rating Life Events Checklist (ASLEC) (46). This scale includes 27 items describing a life event, and the participant selects an option according to his/her own judgment (1 representing “*no impact*” and 5 representing “*extremely serious impact*”). The scale has good properties for validity and reliability (46, 47). In this research, the total scale Cronbach'α coefficient is 0.935.

#### Teachers' Depressive Symptoms

Teachers completed the Center for Epidemiologic Studies Depression Scale (CES-D) (48). The scale is a 20-item self-report measure designed to assess depressive symptoms in the general population. For each item, participants were asked how often they had experienced the symptom in the last week, with responses ranging from 1 (“*rarely or never*”) to 4 (“*very often or always*”). Total scores range from 20 to 80 points, with higher scores indicating higher levels of depressive symptoms. In this research, the total scale Cronbach'α coefficient is 0.902.

#### Teachers' Emotional Labor

Teacher participants completed Emotional Labor Scales for Elementary and Middle School Teachers (ELSEMST) (49). The scale was consisted of four dimensions: emotional perception (13 items), deep acting (8 items), surface acting (9 items), and natural acting (3 items). Each item is scored from 1 (“*very inconsistent*”) to 5 (“*very consistent*”) and a higher score indicates a higher degree of individual emotional labor. The scale has been proven to be of good reliability and validity. The total scale and its four dimensions' test-retest reliability coefficients ranged from 0.84 to 0.92 and Cronbach α values were 0.64~0.85. Based on the criterion of the Positive and Negative Affect Schedule (PANAS) (50), one of the most widely used affect scales (51), it was possible to separate teacher participants into groups comprising as follows: (1) low positive affect (PA), high negative affect (NA); and (2) high positive affect (PA), low negative affect (NA). The groups were used to compare differences between the two types of ELSEMST total scores and the dimensions score. The results showed that ELSEMST had a good criterion validity based on scores from the PANAS (49). In this research, the total scale Cronbach'α coefficient is 0.914.

#### Covariates

We controlled for the following covariates: number of siblings (only-child or child with siblings), age, gender, and the life events of students. These variables have been shown to correlate with depression (34, 52).

### Data Preprocessing and Analysis

#### Computing Weighted Depressive Symptoms in Teachers

We matched teachers' and students' data of the same class. In order to reflect the influence of different disciplines teachers engaged with (e.g., Chinese, mathematics, physics), referred to the studies of Venkatesan et al. (53) and Tsiachristas et al. (54), different weights were assigned based on the Hunan Province (China) compulsory education curriculum (experimental) plan setting table (55) and combined with the results of our interviews with the relevant schools. The weights were: Chinese, Mathematics, and English teachers assigned 0.3 (About 4–6 lessons per week for different school); Physics, Chemistry, Biology, History, Geography, and Politics teachers assigned 0.2 (About 2–4 lessons per week for different school); Music, Painting, and Labor Skills teachers assigned 0.1(About 1–2 lessons per week for different school). The largest weight was 0.4 assigned to the head teacher^1^. The score for teachers' depressive symptoms of the same class is calculated according to the following formula:


(1) $$\begin{array}{r} \text{CTDSWS }=\;0.4\times \text{DSSH}+\frac{0.3\times \sum_{i=1}^{n_{1}}D_{i}}{n_{1}}\\ \;+\frac{0.2\times \sum_{j=1}^{n_{2}}D_{j}}{n_{2}}+\frac{0.1\times \sum_{k=1}^{n_{3}}D_{k}}{n_{3}} \end{array}$$

*CTDSWS: Class teachers' depressive symptom weighted score; DSSH: Depressive symptom score of the headteacher; D*_*i*_*: Depressive symptoms score of a teacher who teaches Chinese, Mathematics, or English courses; D*_*j*_*: Depressive symptoms score of a teacher who teaches Physics, Chemistry, Biology, History, or Geography; D*_*k*_*: Depressive symptoms score of a teacher who teaches Music, Painting, or Labor Skill; n*_1_*, n*_2_*, n*_3_*: Number of the three types of courses. Such as: If a class has only the depressive symptom score of the Chinese teacher, then n*_1_ *is 1. If there is Chinese, Mathematics and English teacher's depressive symptom score, then n*_1_ *is 3. n*_2_ *and n*_3_ *are the same as n*_1_.

#### Computing Weighted Emotional Labor Scores of Teachers

The assignment of weight did not change. The score for teacher's emotional labor of the same class will be calculated according to Formula 2 and Formula 3:


(2) $$\begin{array}{r} \text{CTELSAS }=\;0.4\;\times \text{ ESASH }+\;\frac{0.3\times \sum_{i=1}^{n_{1}}ES_{i}}{n_{1}}\;\\ \;+\;\frac{0.2\times \sum_{j=1}^{n_{2}}ES_{j}}{n_{2}}\;+\;\frac{0.1\times \sum_{k=1}^{n_{3}}ES_{k}}{n_{3}} \end{array}$$

(3) $$\begin{array}{r} \text{CTELDAS }=\;0.4\;\times \text{ EDASH }+\;\frac{0.3\times \sum_{i=1}^{n_{1}}ED_{i}}{n_{1}}\;\\ \;+\;\frac{0.2\times \sum_{j=1}^{n_{2}}ED_{j}}{n_{2}}\;+\;\frac{0.1\times \sum_{k=1}^{n_{3}}ED_{k}}{n_{3}} \end{array}$$

*in Formula 2, CTELSAS: Class teacher' emotional labor surface acting score; ESASH: Emotional labor Surface Acting score of the head teacher; ES*_*i*_*: Emotional Labor Surface Acting Score of a teacher who teaches Chinese, or Mathematics, or English courses; ES*_*j*_*: Emotional Labor Surface Acting Score of a teacher who teaches Physics, Chemistry, Biology, History or Geography; ES*_*k*_*: Emotional Labor Surface Acting Score of a teacher who teaches Music, Painting, or Labor Skills; n*_1_*, n*_2_*, n*_3_*: as for formula 1; in Formula 3, CTELDAS: Class teacher' emotional labor deep acting Score; EDASH: Emotional labor Deep Acting score of the head teacher; ED: Emotional Labor Deep Acting Score of a teacher and the subscript “i, j, k” has the same meaning as formula 2*.

#### Cross-Level Linear Model Analysis

Since the students and teachers are from the same class and form a hierarchical nested structure, the cross-level linear model analysis can be used to analyze the nested data. The teacher is as the upper level of the hierarchical structure and the student is as the lower level of the hierarchical structure. We used Statistical Analysis System (SAS, version 9.4) mixed procedure to analyze: (1) whether the teachers' depressive symptoms significantly affect the students' depressive symptoms; (2) whether the influence of teachers' depressive symptoms on students' depressive symptoms would be moderated or mediated by the students' negative cognition style and dysfunctional attitude; and (3) whether the teachers' emotional labor would moderate the influence of teachers' depressive symptoms on students' depressive symptoms.

## Results

### Descriptive Statistics and Correlations

We calculated the means, standard deviations, and correlation coefficients of the variables. The results showed that students' depressive symptoms were significantly correlated to students' age (*r* = 0.125, *P* < 0.01), life events (*r* = 0.349, *P* < 0.001), teachers' depressive symptom (*r* = −0.075, *P* < 0.01), teachers' emotional labor surface acting (*r* = −0.047, *P* < 0.05), and teachers' emotional labor deep acting (*r* = −0.059, *P* < 0.01); Teachers' depressive symptoms were positively correlated to teachers' emotional labor surface acting (*r* = 0.601, *P* < 0.001) and deep acting (*r* = 0.717, *P* < 0.001). For details, see Table 1.

**Table 1:** The variables' mean, standard deviation and correlation coefficients.

**Variables**	***M (SD)***	**1**	**2**	**3**	**4**	**5**	**6**	**7**
1. Age	13.35 ± 1.01	1						
2. Gender	1.51 ± 0.50	−0.042^*^	1					
3. SN	0.54 ± 0.50	0.091^**^	−0.161^**^	1				
4. T_Events	47.69 ± 26.24	0.090^**^	0.004	−0.075^*^	1			
5. CDI	13.28 ± 7.67	0.125^**^	0.01	−0.033	0.349^***^	1		
6. CTDSWS	36.64 ± 9.03	−0.061^**^	−0.014	−0.064^*^	0.003	−0.075^**^	1	
7. CTELSAS	3.10 ± 0.59	−0.002	0.016	−0.021	−0.033	−0.047^*^	0.601^***^	1
8. CTELDAS	3.57 ± 0.66	−0.042^*^	0.000	−0.005	−0.017	−0.059^**^	0.717^***^	0.841^***^

*SN, Only-child or Siblings; T_Events, Student's life events total score; CDI, Student's depressive symptom total score; CTDSWS, Class teachers' depressive symptom weighted score; CTELS, Class teachers' emotional labor score; CTELSAS, Class teacher' emotional labor surface acting score; CTELDAS, Class teacher' emotional labor deep acting Score. Gender: Male = 1; Female = 2; Both Age and Gender refer to that of the students*.

* *P <0.05*;

** *P <0.01*;

*** *P <0.001*.

### Cross-Level Influencing and Moderating Role

For the multilevel model analysis, the measured outcome variable data should meet the assumption of normal distribution. Therefore, we tested whether the students' depressive symptoms scores adhered to the normal distribution. The results showed that the data did not reflect a normal distribution (see Table 2). We attempted to transform the non-normal data using two common methods: log transformation and square-root transformation (56). We tested the skewness and kurtosis coefficients of the original data and transformed data; the results showed that only the square-root transformed data met the requirement of the normal distribution since the 95% confidence interval of skewness and kurtosis coefficients included zero. Thus, we utilized the data transformed by square-root method for further analysis.

**Table 2:** Students' depressive symptoms data normality tests.

**Data type**	**Coefficient**	**Statistic**	**Std. error**	**Bootstrap**			
				**Bias**	**Std. error**	**95% confidence interval**	
						**Lower**	**Upper**
Original	*Skewness*	0.920	0.048	−0.004	0.087	0.746	1.092
	*Kurtosis*	1.430	0.097	−0.024	0.422	0.602	2.276
Square-root	*Skewness*	0.079	0.048	−0.001	0.046	−0.012	0.170
	*Kurtosis*	−0.197	0.097	−0.002	0.098	−0.382	0.002
Base-10 Log	*Skewness*	−0.881	0.048	0.001	0.050	−0.979	−0.780
	*Kurtosis*	0.965	0.097	−0.003	0.150	0.672	1.265

*Bootstrap results based on 6,000 bootstrapping samples*.

Following Wang et al.'s approach (57), we fitted an empty model and then examined the cross-level effects of teachers' depression on students' depression.

*Empty Model:*


(4) $$\begin{array}{r} \text{Student level}:\;\sqrt{CDI_{ij}\;}={\;\beta }_{0j}+\varepsilon_{ij} \end{array}$$

(5) $$\begin{array}{r} \text{Teacher level}:{\;\beta }_{0j}\;=\;\gamma_{00}+\mu_{0j} \end{array}$$

The output of the SAS restricted maximum likelihood (REML) estimation showed that the variance in the student level random intercept coefficients and the residual variance were as follows: ${\hat{\sigma }}_{\mu 0}^{2}=0.059$ (*P* < 0.001), and ${\hat{\sigma }}^{2}=1.081$ (*P* < 0.001)


(6) $$\begin{array}{r} ICC=\frac{{\hat{\sigma }}_{\mu 0}^{2}}{{\hat{\sigma }}_{\mu 0}^{2}+{\hat{\sigma }}^{2}}=\frac{0.059}{0.059+1.081}=0.052 \end{array}$$

(7) $$\begin{array}{r} deff\;=\;1+\left(groupmean-1\right)\times ICC=1+\left(23-1\right)\\ \;\times 0.052=2.144 \end{array}$$

As ${\hat{\sigma }}_{\mu 0}^{2}$ is statistically significant (*P* < 0.001), we concluded that the intraclass correlation coefficient (*ICC)* was statistically significant. In addition, since the design effect (*deff* ) was over 2, a multilevel model is necessary for data analysis (57, 58).

#### The Cross-Level Influence of Teachers' Depression on the Students' Depression

According to the suggestions of Aguinis et al. (59), in order to improve the interpretation of results when conducting multilevel analyses, we centered level 1 continuous variables by group mean and centered those of level 2 by grand mean. We then analyzed the cross-level influence, the teachers' depressive symptoms in level 2 as predictive variable, and the students' depressive symptoms as a dependent variable. In order to make the cross-level prediction more accurate, we set gender, age, siblings (only-child or child with siblings), and student life events as control variables. The model produced was as follows:

Student level:


(8) $$\begin{array}{r} \sqrt{CDI_{ij}}\;=\;\beta_{0j}\;+\;\beta_{1j}\times gender\;+\;\beta_{2j}\;\times \;age\;+{\;\beta }_{3j}\;\\ \;\times \;SN\;+\;\beta_{4j}\;\times \;T\text{\_}Events\;+\;\varepsilon_{ij} \end{array}$$

(*Note: SN: siblings; T_Events: total score of student life events*.)

The teacher level:


(9) $$\begin{array}{r} \beta_{0j}\;=\;\gamma_{00}\;+\;\gamma_{01}\text{CTDSWS }+\;\mu_{0j} \end{array}$$

Substituting Equation (9) into Equation (8) leads to combined model (10):


(10) $$\begin{array}{r} \;\sqrt{CDI_{ij}}\;=\;\beta_{1j}\times T\text{\_}Events+\beta_{2j}\times age+\beta_{3j}\\ \times gender+\beta_{4j}\times SN+\gamma_{00}+\gamma_{01}\text{CTDSWS}+\mu_{0j}+\varepsilon_{ij} \end{array}$$

The results (see Table 3) show that the teachers' depressive symptoms significantly predicted students' depressive symptom (β = −0.089, *Se* = 0.031, *F* = 8.38, *P* = 0.005 <0.01). However, to our surprise, this prediction completely reversed our hypothesis. The regression coefficient of teachers' depression in the predictive model is a negative value (β = −0.089), which indicates that when the teacher's depressive symptoms were increasing, the students' depressive symptoms were decreasing. Because the study's first hypothesis was that teachers' depression can transmit to students was not supported by our data analysis, we did not explore the cross-level interaction of students' cognitive factors and teachers' depressive symptoms further. After skipping the verification of Hypothesis 2, we proceeded to examine Hypothesis 3 concerning the potential moderating effect of emotional labor.

**Table 3:** Cross-level analysis for the multilevel model.

**Variables**	**β**	***Se***	***df***	***F***	***P***
T_Events	0.015	0.001	2,185	317.29	<0.001
Age	0.118	0.033	2,185	12.60	<0.001
Gender	0.045	0.042	2,185	1.19	0.275
SN	−0.071	0.044	2,185	2.66	0.103
CTDSWS	−0.089	0.031	109	8.38	0.005

### The Moderating Role of Teachers' Emotional Labor

In order to explore the moderating role of emotional labor more clearly, we analyzed the two executive strategies of emotional labor: surface acting and deep acting. The results of the SAS mixed analysis are shown in Table 4. The interaction of teachers' depression with emotional labor surface acting was not significant (β = −0.075, *Se* = 0.048, *F* = 2.41, *P* = 0.123). However, the interaction between teachers' depression and teachers' emotional labor deep acting was significant (β = −0.016, *Se* = 0.009, *F* = 4.07, *P* = 0.046 <0.05).

**Table 4:** Cross-level interaction analysis of the multilevel model with emotional labor as the moderating variable.

**Variables**	**β**	***Se***	***df***	***F***	***P***	**Variables**	**β**	***Se***	***Df***	***F***	***P***
(1) T_Events	0.382	0.020	2,355	356.56	<0.001	(1) T_Events	0.382	0.020	2,355	355.69	<0.001
(2) Grade	0.025	0.039	107	0.43	0.515	(2) Grade	0.026	0.039	107	0.45	0.505
(3) CTELSAS	−0.001	0.069	107	0.00	0.989	(3) CTELDAS	−0.076	0.075	107	1.01	0.316
(4) CTDSWS	0.114	0.141	107	0.65	0.423	(4) CTDSWS	0.214	0.154	107	1.93	0.168
(3) × (4)	−0.075	0.048	107	2.41	0.123	(3) × (4)	−0.016	0.009	107	4.07	0.046

*CTELSAS represents class teacher' emotional labor surface acting score; CTELDAS represents class teacher' emotional labor deep acting score*.

**Figure 1:**
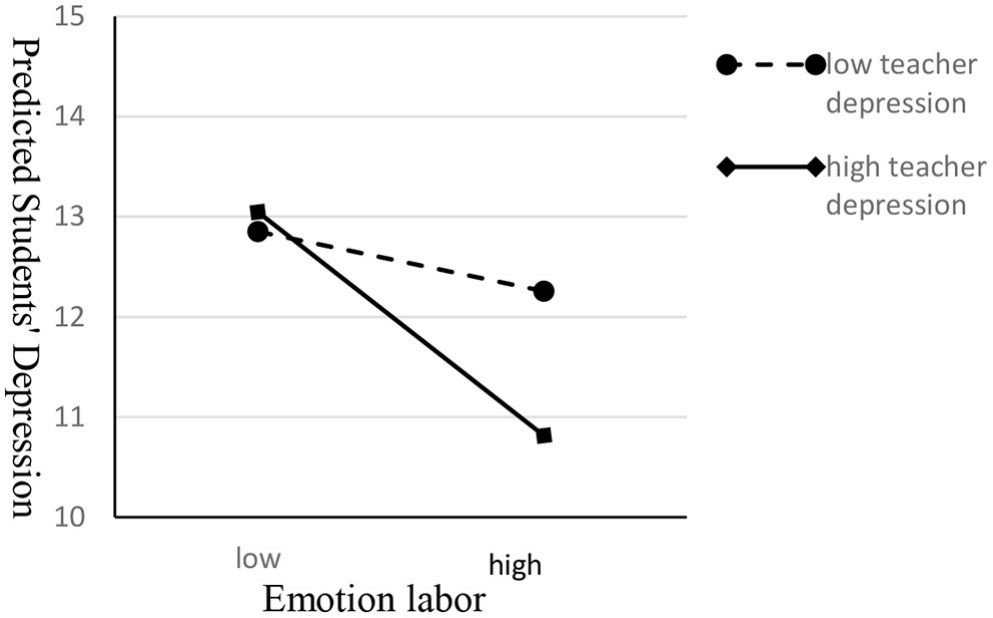
The interaction between teachers' depressive symptom and deep acting of emotional labor. As the root squared values of the students' depressive symptoms was used as the dependent variable, we squared the predictive values of the students' depressive symptoms.

To further clarify the interaction CTELDAS × CTDSWS, using a SAS mixed estimation procedure, we estimated the students' depression score under the conditions of high teachers' depression vs. low teacher' depression (plus or minus 1 between-class standard deviation) and high emotional labor deep acting vs. low emotional labor deep acting (plus or minus 1 between-class standard deviation). The results show that (see Figure 1): for teachers with high levels of depressive symptoms, their emotional labor deep acting significantly affects the influence of teachers' depression on students' depression [*Slope* = −0.162, *Se* = 0.054, *t* (107) = −3.01, *P* = 0.003 <0.01]. However, for teachers with low depressive symptoms, their emotional labor deep acting did not significantly affect the influence of teachers' depression on students' depression [*Slope* = −0.042, *Se* = 0.052, *t* (107) = −0.81, *P* = 0.422]. Comparing emotional labor deep acting of teachers with high depressive symptoms to those of teachers with low depressive symptoms, the former had a considerably larger effect on the influence of teachers' depression on students' depression [*Slope* = −0.120, *Se* = 0.059, *t* (107) = 2.03, *P* = 0.044 <0.05].

## Discussion

### Does Teachers' Depression Transmit to Their Students?

According to the theory of emotional contagion (30), we hypothesized that teachers' depression would transmit to their students and that students' vulnerable cognitions, such as negative cognition style and dysfunctional attitude, might act as moderating or mediating variables in this process. However, the study results showed that teachers' depressive symptoms, cross-level, inversely predicted the students' depressive symptoms. This finding indicates that the higher teachers' depressive symptoms, the lower the students' depressive symptoms. The results were directly contrary to our hypothesis and did not conform to the theory of emotional contagion. We suggest four reasons for this. First, teachers not only impart knowledge to students, but also have a responsibility to ensure that their students develop healthily (60, 61). For this reason, most teachers with depressive symptoms would be likely to inhibit their depressive emotions while communicating with their students, thereby blocking the contagions of their depression to students.

Second, teachers with higher levels of depressive symptoms may reduce their involvement in work-related activities, which would reduce time spent with students. Therefore, the influence of their depression on their students would be decreased. Although there are few studies concerning the association between teachers' depression and their teaching activities, they consistently show that teachers who experience higher levels of depressive symptoms have decreased sensitivity and greater withdrawal in terms of interactions with their students (28, 62, 63).

Third, the secondary schools in China, teachers' work performance is primarily assessed on the student achievement. In order to achieve their own better teaching performance, the teachers with low depressive symptoms may spend more time encouraging their students to study, assigning students more homework, and more stringently requiring for students. This, however, might increase the stress experienced by the students, thereby increasing their depressive symptoms. Huang, Wu, Hu, and Yang found that teachers' emotional overinvolvement had a significant effect on students' depression (64).

Fourth, Moses took adolescents with diagnosed mental disorders as participants, he found that one-third reported perceiving stigma from school teachers; for example, they believe that school teachers feared, disliked, or avoided them (65), students who perceive stigma by others were less likely to seek support and receive services (66), this might deteriorate their depressive symptom. However, teachers with depression may be more empathetic to students' depression, and take deep acting of emotional labor to help depression students, thus reducing the likelihood of stigmatization of it, they also more possibly take deep acting of emotional labor to help depression students, further decrease students' depression. Considering that our study indicates that teachers' depression is not transmitted to students, we did not further verify the mediating or moderating role of students' depressive cognitive vulnerable factors.

### The Moderating Role of Teachers' Emotional Labor

In order to verify the third hypothesis of the study, we analyzed the moderating role of the teachers' emotional labor. The results showed that teachers' deep acting of emotional labor significantly affected the influence of teachers with high depression symptoms on students' depression symptoms. However, for the teachers with low depression symptoms, the influence of teachers' emotional labor was not significant (for detail, please see Figure 1). This result may illustrate that teachers' emotional labor deep acting blocks the contagion of their depression to students and potentially alleviates the influence of teachers' high levels of depressive symptoms on students. Hennig-Thurau et al. found that the employees' deep acting emotional labor influenced the customers' emotions and perceptions (67). In our study, we further found that the deep acting of emotional labor significantly moderated the influence of teachers' depression on students, but there is no significant interaction between the surface acting of emotional labor and the depressive symptoms of teachers. In the field of sales, Seger-Guttmann and Medler-Liraz had the similarly finding, they found even when customers were highly involved in the purchasing process, they spent less money when they observed employee inauthenticity as manifested in Surface Acting. However, Deep Acting positively moderated the relationship between customer participation and spending money (68). Although the study found that deep acting could moderate the influence of teachers' depression on students' depression, both deep acting and surface acting were positively correlated with teachers' depression significantly on the class level, meaning that emotional labor may be a double-edged sword: while buffering the impact of teachers' depression on students' depression, it will also inversely affect teachers' own depression negatively. As there are few studies on teachers' emotional labor and teachers' depression, further research is needed for confirmation.

### Limitation

Using the nested model of class teachers and students, the study explored whether teachers' depression would be transmitted to students. As far as we know, maybe this is the first study focus on this issue. For this reason, it is inevitable that there are some limitations for this study. First, as all the data were self-reported and cross-sectional, causal associations cannot be identified. Second, whether teachers' depression would be contagious to students, largely depends on their contacting time, we just us class schedule as the basis of judging contacting time between teachers and students, and assign different weight to different discipline teacher, it may not reflect the real state. Third, From the research results, the influence of teachers' depression on students, and the moderating role of teachers 'emotional labor, may be related to teachers' professional ethics and school management. however, our investigation did not involve these contents, it makes us difficult to exactly answer some questions, such as why teachers' depression negatively affects students' depression. further study should consider the factors related to teachers' vocational ethics and teachers' management.

## Conclusion

The depression of teachers does not appear to be not readily transmitted to their students. One of reasons may be that the deep acting of teachers' emotional labor play a role of alleviating the effect of higher levels of depressive symptoms on their students. However, the emotional labor surface acting and deep acting are positively correlated with teachers' depression, predicting that teachers' emotional labor may unsustainable. Considering this, better results may be generated for students' depression interventions based in school by including teachers in the intervention programs.

## Code Availability Statement


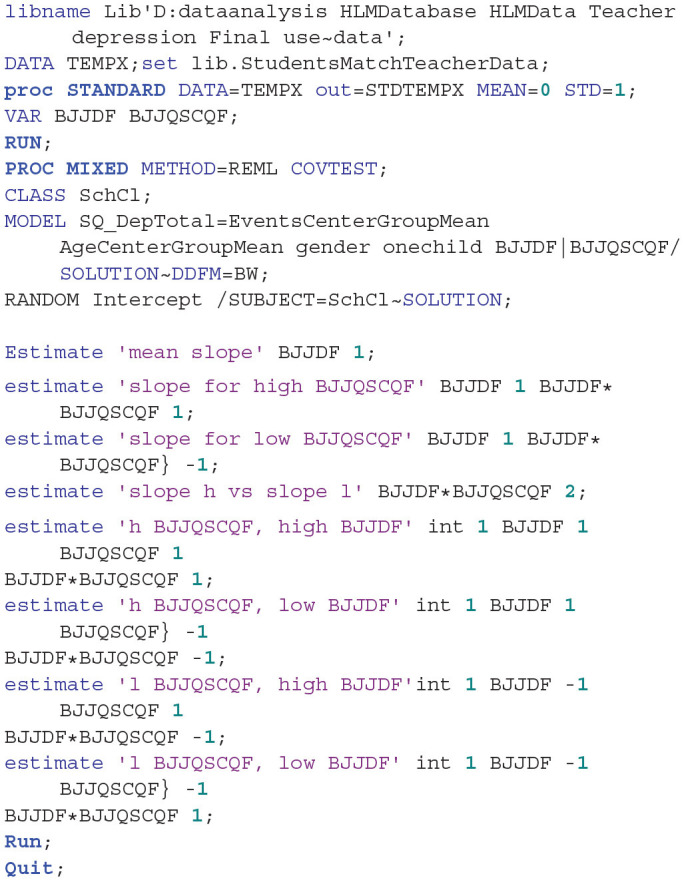


## Data Availability Statement

The raw data supporting the conclusions of this article will be made available by the authors, without undue reservation.

## Ethics Statement

The studies involving human participants were reviewed and approved by Research Ethical Committee of Guizhou Normal University Psychological School. Written informed consent to participate in this study was provided by the participants self or the participants' legal guardian/next of kin.

## Author Contributions

WW was involved in the design, conduct, and analysis and writing of this paper. YL was involved in the data collection and analysis. Both authors contributed to the article and approved the submitted version.

## Funding

This study was supported by Science and Technology Plan Project of Guizhou Province (黔科合基础 [2019]1240号), by Humanities and Social Science Research Program of Ministry of Education of China (20YJA190006), and funded by Funding Projects of Ph.D. Research in Guizhou Normal University for WW (0516019). The funders had no role in study design, data collection and analysis, decision to publish, or preparation of the manuscript.

## Conflict of Interest

The authors declare that the research was conducted in the absence of any commercial or financial relationships that could be construed as a potential conflict of interest.

## Publisher's Note

All claims expressed in this article are solely those of the authors and do not necessarily represent those of their affiliated organizations, or those of the publisher, the editors and the reviewers. Any product that may be evaluated in this article, or claim that may be made by its manufacturer, is not guaranteed or endorsed by the publisher.

## Footnotes

^1^Head teacher: A teacher who is not only responsible for the teaching of class courses (usually the main subjects such as language or mathematics), but also the daily management of class students.

## Abbreviations

CDI: Children's Depression Inventory
CCSQ: Children's Cognitive Style Questionnaire
CDAS: Children's Dysfunctional Attitudes Scale
ASLEC: Adolescent Self-Rating Life Events Checklist
CES-D: Center for Epidemiologic Studies Depression Scale
ELSEMST: Emotional Labor Scales for Elementary and Middle School Teachers
CTDSWS: Class teachers&#8217
depressive symptom weighted score
DSSH: Depressive symptom score of the head teacher
CTELSAS: Class teacher&#8217
emotional labor surface acting score
ESASH: Emotional labor surface acting score of the head teacher
CTELDAS: Class teacher&#8217
emotional labor deep acting Score
EDASH: Emotional labor deep acting score of the head teacher
ICC: Intraclass Correlation Coefficient
DEFF: Design effect
REML: Restricted Maximum Likelihood.

## References

[1] Kazdin AE. Annual research review: expanding mental health services through novel models of intervention delivery. J Child Psychol Psychiatry. (2019) 60:455–72. 10.1111/jcpp.12937 29900543

[2] De Girolamo G Dagani J Purcell R Cocchi A McGorry P. Age of onset of mental disorders and use of mental health services: needs, opportunities and obstacles. Epidemiol Psychiatr Sci. (2012) 21:47–57. 10.1017/S2045796011000746 22670412

[3] Kessler RC Wang PS. The descriptive epidemiology of commonly occurring mental disorders in the United States. Annu Rev Public Health. (2008) 29:115–29. 10.1146/annurev.publhealth.29.020907.090847 18348707

[4] Polanczyk GV Salum GA Sugaya LS Caye A Rohde LA. Annual research review: a meta-analysis of the worldwide prevalence of mental disorders in children and adolescents. J Child Psychol Psychiatry. (2015) 56:345l disori: 10.1111/jcpp.12381 25649325 10.1111/jcpp.12381

[5] Bruha L Spyridou V Forth G Ougrin D. Global child and adolescent mental health: challenges and advances. Lond J Primary Care. (2018) 10:108–9. 10.1080/17571472.2018.1484332

[6] Merikangas KR Nakamura EF Kessler RC. Epidemiology of mental disorders in children and adolescents. Dialog Clin Neurosci. (2009) 11:7–20. 10.31887/DCNS.2009.11.1/krmerikangas 19432384 PMC2807642

[7] Johnstone KM Kemps E Chen J. A meta-analysis of universal school-based prevention programs for anxiety and depression in children. Clin Child Fam Psychol Rev. (2018) 21:466–81. 10.1007/s10567-018-0266-5 30105480

[8] Bor W Dean AJ Najman J Hayatbakhsh R. Are child and adolescent mental health problems increasing in the 21st century? A systematic review. Austral N Z J Psychiatry. (2014) 48:606–16. 10.1177/0004867414533834 24829198

[9] Thapar A Collishaw S Pine DS Thapar AK. Depression in adolescence. Lancet. (2012) 379:1056–67. 10.1016/S0140-6736(11)60871-4 22305766 PMC3488279

[10] Li Y-P Ma G Schouten EG Hu X Cui Z Wang D. Report on childhood obesity in China (5) body weight, body dissatisfaction and depression symptoms of Chinese children aged 9-10 years. Biomed Environ Sci. (2007) 20:11–8. 10.1016/j.biocon.2006.08.028 17458136

[11] Guo L Deng J He Y Deng X Huang J Huang G. Prevalence and correlates of sleep disturbance and depressive symptoms among Chinese adolescents: a cross-sectional survey study. BMJ Open. (2014) 4:e5517. 10.1136/bmjopen-2014-005517 25079937 PMC4120320

[12] Hong X Li J Xu F Tse LA Liang Y Wang Z. Physical activity inversely associated with the presence of depression among urban adolescents in regional China. BMC Public Health. (2009) 9:148. 10.1186/1471-2458-9-148 19457241 PMC2693135

[13] Yang J Yao S Zhu X Zhang C Ling Y Abela JR. The impact of stress on depressive symptoms is moderated by social support in Chinese adolescents with subthreshold depression: a multi-wave longitudinal study. J Affect Disord. (2010) 127:113–21. 10.1016/j.jad.2010.04.023 20554013

[14] Li J Chen X Zhao C Xu Y. Prevalence of depression in Chinese children and adolescents: a meta-analysis. Chin J Child Health Care. (2016) 24:295–8. 10.11852/zgetbjzz2016-24-03-22

[15] Hopkins K Crosland P Elliott N Bewley S. Diagnosis and management of depression in children and young people: summary of updated NICE guidance. BMJ. (2015) 350:h824. 10.1136/bmj.h824 25739880

[16] Weisz JR McCarty CA Valeri SM. Effects of psychotherapy for depression in children and adolescents: a meta-analysis. Psychol Bull. (2006) 132:132–49. 10.1037/0033-2909.132.1.132 16435960 PMC2150594

[17] Prinzie P van Harten LV Deković M van den Akker AL Shiner RL. Developmental trajectories of anxious and depressive problems during the transition from childhood to adolescence: Personality × Parenting interactions. Dev Psychopathol. (2014) 26:1077–92. 10.1017/S0954579414000510 24914625

[18] Wolpert M., Zamperoni, V., Napoleone, E., Patalay, P., Jacob, J., & Fokkema, M., et al. Predicting mental health improvement and deterioration in a large community sample of 11-to 13-year-olds. Eur Child Adolesc Psychiatry. (2020) 29:167–78. 10.1007/s00787-019-01334-4 31054126 PMC7024693

[19] Brunwasser SM Freres DR Gillham JE. Youth cognitive-behavioral depression prevention: testing theory in a randomized controlled trial. Cogn Ther Res. (2018) 42:468–82. 10.1007/s10608-018-9897-6 30057434 PMC6059657

[20] Brunwasser SM Garber J. Programs for the prevention of youth depression: evaluation of efficacy, effectiveness, and readiness for dissemination. J Clin Child Adolesc Psychol. (2016) 45:763–83. 10.1080/15374416.2015.1020541 25933173 PMC5176361

[21] Calear AL Christensen H. Systematic review of school-based prevention and early intervention programs for depression. J Adolesc. (2010) 33:429–38. 10.1016/j.adolescence.2009.07.004 19647310

[22] Sutton JM. Prevention of depression in youth: a qualitative review and future suggestions. Clin Psychol Rev. (2007) 27:552–71. 10.1016/j.cpr.2007.01.014 17355898 PMC1952210

[23] Miller L Musci R D'Agati D Alfes C Beaudry MB Swartz K. Teacher mental health literacy is associated with student literacy in the Adolescent Depression Awareness Program. School Ment Health. (2019) 11:357–63. 10.1007/s12310-018-9281-4 31579430 PMC6774623

[24] Blossom JB Adrian MC Vander Stoep A McCauley E. Mechanisms of change in the prevention of depression: an indicated school-based prevention trial at the transition to high school. J Am Acad Child Adolesc Psychiatry. (2020) 59:541–51. 10.1016/j.jaac.2019.05.031 31228560 PMC6920576

[25] Spence SH Shortt AL. Research Review: can we justify the widespread dissemination of universal, school-based interventions for the prevention of depression among children and adolescents? J Child Psychol Psychiatry. (2007) 48:526–42. 10.1111/j.1469-7610.2007.01738.x 17537069

[26] Werner-Seidler A Perry Y Calear AL Newby JM Christensen H. School-based depression and anxiety prevention programs for young people: a systematic review and meta-analysis. Clin Psychol Rev. (2017) 51:30–47. 10.1016/j.cpr.2016.10.005 27821267

[27] Jennings PA Greenberg MT. The prosocial classroom: teacher social and emotional competence in relation to student and classroom outcomes. Rev Educ Res. (2009) 79:491–525. 10.3102/0034654308325693

[28] Granger K. Promoting High Quality Teacher-Child Interactions: Examining the Role of Teachers' Depression, Perceptions of Children's Peer Relationships, and Contextual Factors. Arizona State University (2017).

[29] Birchwood M. Pathways to emotional dysfunction in first-episode psychosis. Brit J Psychiatry. (2003) 182:373–5. 10.1192/bjp.182.5.373 12724236

[30] Hill AL Rand DG Nowak MA Christakis NA. Emotions as infectious diseases in a large social network: the SISa model. Proc R Soc B Biol Sci. (2010) 277:3827–35. 10.1098/rspb.2010.1217 20610424 PMC2992714

[31] Bastiampillai T Allison S Chan S. Is depression contagious? The importance of social networks and the implications of contagion theory. Austral N Z J Psychiatry. (2013) 47:299–303. 10.1177/0004867412471437 23568155

[32] Joiner Jr TE Katz J. Contagion of depressive symptoms and mood: Meta-analytic review and explanations from cognitive, behavioral, and interpersonal viewpoints. Clin Psychol Sci Pract. (1999) 6:149–64. 10.1093/clipsy.6.2.149

[33] Tee EY. The emotional link: Leadership and the role of implicit and explicit emotional contagion processes across multiple organizational levels. Leadersh Q. (2015) 26:654–70. 10.1016/j.leaqua.2015.05.009

[34] Abela JR Hankin BL. Handbook of Depression in Children and Adolescents. New York: The Guilford Press (2008).

[35] Hankin BL. Future directions in vulnerability to depression among youth: Integrating risk factors and processes across multiple levels of analysis. J Clin Child Adolesc Psychol. (2012) 41:695–718. 10.1080/15374416.2012.711708 22900513 PMC4030594

[36] Kertz SJ Petersen DR Stevens KT. Cognitive and attentional vulnerability to depression in youth: a review. Clin Psychol Rev. (2019) 71:63–77. 10.1016/j.cpr.2019.01.004 30732975

[37] Lu Y Li B Zhan L Wu W. Life events and middle school students' depression: the moderating role of class teacher's emotional labor. Chin J Clin Psychol. (2014) 22:885–8.

[38] Hochschild Arlie R. The Managed Heart: Commercialization of Human Feeling. Berkeley, CA, University of California Press (1983).

[39] Lu Y Wu W Mei G Zhao S Zhou H Li D. Surface acting or deep acting, who need more effortful? A study on emotional labor using functional near-infrared spectroscopy. Front Hum Neurosci. (2019) 13:151. 10.3389/fnhum.2019.00151 31133836 PMC6524537

[40] Grandey AA Sayre GM. Emotional labor: regulating emotions for a wage. Curr Dir Psychol Sci. (2019) 28:131–7. 10.1177/0963721418812771

[41] Yin H Huang S Chen G. The relationships between teachers' emotional labor and their burnout and satisfaction: a meta-analytic review. Educ Res Rev. (2019) 28:100283. 10.1016/j.edurev.2019.100283

[42] Kovacs M. Children's Depression Inventory, Manual Update. New York, NY: Multi-Health Systems Inc. (2003).

[43] Wu W Lu Y Tan F Yao S. Reliability and validity of the Chinese version of children's depression inventory. Chin Mental Health J. (2010) 24:775–9. 10.3969/j.issn.1000-6729.2010.10.014

[44] Abela JR. The hopelessness theory of depression: a test of the diathesis-stress and causal mediation components in third and seventh grade children. J Abnorm Child Psychol. (2001) 29:241–54. 10.1023/A:1010333815728 11411786

[45] Abela JR Sullivan C. A test of Beck's cognitive diathesis-stress theory of depression in early adolescents. J Early Adolesc. (2003) 23:384–404. 10.1177/0272431603258345

[46] Liu X Liu L Yang J Cai F Wang A Sun L. The reliability and validity analysis of the adolescent self-rating life events check list. Chin J Clin Psychol. (1997) 5:34–6.28006677

[47] Xin X Yao S. Validity and reliability of the adolescent self-rating life events checklist in middle school students. Chin Mental Health J. (2015) 29:355–60. 10.3969/j.issn.1000-6729.2015.05.010

[48] Radloff LS. The CES-D scale: a self-report depression scale for research in the general population. Appl Psychol Meas. (1977) 1:385–401. 10.1177/01466216770010030623302475

[49] Wu YJ Liu Y. Development of emotional labor scale for elementary and secondary school teachers. J Northwest Normal Univ. (2011) 48:102–8. 10.3969/j.issn.1001-9162.2011.01.01731905608

[50] Watson D Clark LA Tellegen A. Development and validation of brief measures of positive and negative affect: the PANAS scales. J Pers Soc Psychol. (1988) 51:1063–70. 10.1037/0022-3514.54.6.1063 3397865

[51] Seebach CL Kirkhart M Lating JM Wegener ST Song Y Riley LH III. Examining the role of positive and negative affect in recovery from spine surgery. Pain. (2012) 153:518–25. 10.1016/j.pain.2011.10.012 22119337

[52] Aitang L Yunqiang Y Hongsheng L. Anxiety/depression of plateau forces only child officers and men. China J Health Psychol. (2012) 27:984−6.

[53] Venkatesan G Fragomeni S Zhang G. An investigation into the student grading system in problem based learning. Paper presented at the Proceedings of the 2008 European Society for Engineering Education International Annual Conference. Rotterdam (2008).

[54] Tsiachristas A Cramm JM Nieboer A Rutten-van Mölken M. Broader economic evaluation of disease management programs using multi-criteria decision analysis. Int J Technol Assess Health Care. (2013) 29:301–8. 10.1017/S0266462313000202 23759317

[55] Hunan Provincial Department of Education. Notice on Printing and Distributing the Hunan province Compulsory Education Curriculum (Experimental) Plan. (2007) Available online at: http://jyt.hunan.gov.cn/sjyt/xxgk/tzgg/201701/t20170120_3950483.html (accessed April 4, 2007).

[56] McDonald JH. Hand book of BioStatistic. Baltimore, MD: Sparky House Publishing (2014).

[57] Wang J Xie H Fisher JH. Multilevel Models: Applications Using SAS. Berlin: Walter de Gruyter (2012). 10.1515/9783110267709

[58] Muthen BO Satorra A. Complex sample data in structural equation modeling. Sociol Methodol. (1995) 25:267–316. 10.2307/271070

[59] Aguinis H Gottfredson RK Culpepper SA. Best-practice recommendations for estimating cross-level interaction effects using multilevel modeling. J Manage. (2013) 39:1490–528. 10.1177/0149206313478188

[60] Lauermann F. Teacher responsibility from the teacher's perspective. Int J Educ Res. (2014) 65:75–89. 10.1016/j.ijer.2013.09.005

[61] Greif Green J Levine RS Oblath R Corriveau KH Holt MK Albright G. Pilot evaluation of preservice teacher training to improve preparedness and confidence to address student mental health. Evid Based Pract Child Adolesc Mental Health. (2020) 5:42–52. 10.1080/23794925.2020.1727793

[62] Jeon L Buettner CK Snyder AR. Pathways from teacher depression and child-care quality to child behavioral problems. J Consult Clin Psychol. (2014) 82:225–35. 10.1037/a0035720 24447005

[63] Whitaker RC Dearth-Wesley T Gooze RA. Workplace stress and the quality of teacher-children relationships in Head Start. Early Child Res Q. (2015) 30:57–69. 10.1016/j.ecresq.2014.08.00829145942

[64] Huang FF Wu CC Hu CY Yang SS. Teacher overinvolvement and student depression among junior high school students in Taiwan. Sci World J. (2006) 6:834–46. 10.1100/tsw.2006.152 16862352 PMC5917210

[65] Moses T. Being treated differently: Stigma experiences with family, peers, and school staff among adolescents with mental health disorders. Soc Sci Med. (2010) 70:985–93. 10.1016/j.socscimed.2009.12.022 20122768

[66] Gulliver A Griffiths KM Christensen H. Perceived barriers and facilitators to mental health help-seeking in young people: a systematic review. BMC Psychiatry. (2010) 10:113. 10.1186/1471-244X-10-113 21192795 PMC3022639

[67] Hennig-Thurau T Groth M Paul M Gremler DD. Are all smiles created equal? How emotional contagion and emotional labor affect service relationships. J Market. (2006) 70:58–73. 10.1509/jmkg.70.3.58

[68] Seger-Guttmann T Medler-Liraz H. Does emotional labor moderate customer participation and buying? Serv Indus J. (2016) 36:356–73. 10.1080/02642069.2016.1219724

